# “Compatibilization” through Elongational Flow Processing of LDPE/PA6 Blends

**DOI:** 10.3390/ma11122375

**Published:** 2018-11-26

**Authors:** Maria Chiara Mistretta, Marco Morreale, Luigi Botta, Manuela Ceraulo, Paolo Fontana, Francesco Paolo La Mantia

**Affiliations:** 1 Department of Civil, Environmental, Aerospace, Materials Engineering, University of Palermo, Viale delle Scienze, 90128 Palermo, Italy; mariachiara.mistretta@unipa.it (M.C.M.); luigi.botta@unipa.it (L.B.); paolofontana2002@virgilio.it (P.F.); 2 Faculty of Engineering and Architecture, Kore University of Enna, Cittadella Universitaria, 94100 Enna, Italy; marco.morreale@unikore.it; 3 UdR INSTM Palermo, Viale delle Scienze, 90128 Palermo, Italy; manuela.ceraulo@gmail.com

**Keywords:** polymer blends, compatibilization, processing, elongational flow

## Abstract

Polyamide/polyolefin blends have gained attention from the academia and the industry for several years. However, in order to optimize their properties, some drawbacks such as chemical incompatibility must be adequately overcome. This can be done by adding suitable compatibilizers. On the other hand, it is less known that suitable processing techniques may also lead to significant results. In a previous work on a low-density polyethylene/polyamide 6 (LDPE/PA6) blend, we found that the orientation due to elongational flow processing conditions could lead to an unexpected brittle–ductile transition. In this work, this phenomenon was further investigated and the attention was mainly focused on the effects that processing can have on the morphology and, as a consequence, on the final properties of a polymer blends. With regard to LDPE/PA6 blend, an important result was found, i.e., the effects on the ductility induced by the elongational flow orientation are similar to those obtained by using an ethylene-glycidyl methacrylate compatibilizer.

## 1. Introduction

Polyamide/polyolefin blends are widely investigated in the literature because of the interesting properties they can show, especially in the view of commercial applications, thanks to the possibility to produce materials with tailored features, without the need of designing new synthesis polymers. At the same time, it is also known that these blends are chemically incompatible, thus with poor physical properties. In addition, significantly different behaviors can be observed, depending on the polyolefin and polyamide type used, their reciprocal amounts, the processing conditions and the presence of additives [1]. In any case, a typical consequence of the incompatibility can be observed on ductility, which is strongly reduced; this occurs since the poor adhesion between the two phases constitutes a defect, which can therefore propagate in presence of mechanical stresses and lead to premature breaking of the sample. This makes compatibilization necessary in order to achieve adequate properties [2,3,4,5,6,7,8,9].

In a previous work [10] we investigated the effect of the orientation (due to elongational flow) on the mechanical properties of a LDPE/PA6 incompatible blend. Although a decrease of elongation at break upon increasing the orientation degree was expected [11,12], the direct comparison between oriented (anisotropic) and unoriented (isotropic) blend samples showed an unexpected, orientation-induced brittle–ductile transition, which was interpreted in terms of morphological changes.

Based on this finding, we deepened the understanding of the behavior of polyamide/polyolefin blends. This work aims at assessing if the effect induced by orientation (and thus by processing) can be compared to that induced by compatibilization. An important finding was obtained, i.e., the importance of processing that, modifying the morphology through elongational flow, can lead to unexpected results: it was shown that (at least for this blend) the action due to the elongational flow leads to effects, which are similar to those obtained by using a compatibilizer. Indeed, the effect of the orientation in an incompatible blend on the elongation at break is very similar to that obtained by adding an ethylene-glycidyl methacrylate compatibilizer. To the best of our knowledge, there is no systematic study in the literature reporting similar conclusions, thus indicating that a novel result was found: “compatibilization” through elongational flow processing of LDPE/PA6 blends can be a very promising and environment-friendly approach; furthermore, it could provide a starting point for investigations on other polymeric blends, thus paving the way for new, alternative routes to chemical compatibilization strategies.

## 2. Materials and Methods

The polymer blends investigated in this work were prepared using a polyamide 6 (PA6) sample (Radilon S35 100 NAT, Radicinova, Bergamo, Italy), having intrinsic viscosity (measured in sulfuric acid) = 3.4 dL/g, and a low density polyethylene (LDPE), film blowing grade sample (FC39, Versalis, San Donato Milanese, Italy; density = 0.923 g/cm^3^, MFI = 0.25 g/10 min at 190 °C and 2.16 kg). The blend was prepared choosing a 75:25 wt/wt LDPE/PA6 ratio (PA6 previously subjected to double-stage exsiccation in ventilated and vacuum oven in order preventing hydrolytic chain scission phenomena during the following processing) and performing the first processing in a corotating twin-screw extruder (OMC, Italy; thermal profile: 140/160/180/200/220/240 °C, screw speed: 200 rpm, output: ~2 kg/h).

Compatibilized blends were obtained in the same way, adding a 5 wt % amount of an ethylene-glycidyl methacrylate copolymer (glycidyl methacrylate content: 8 wt %) commercially available as “Lotader AX 8840” (Arkema, Colombes, France; melt index = 5 g/10 min at 190 °C and 2.16 kg; density = 0.94 g/cm^3^, as reported from the technical data sheet).

For the following characterizations, isotropic samples (10 mm/90 mm/~0.6 mm) were prepared by cutting them off from compression-molded sheets, obtained using the granules coming from the twin-screw extruder and processing them by means of a Carver (Wabash, IN, USA) laboratory press (at 240 °C for approx. 3 min at a ~100 bar pressure). In particular, the sheets were obtained with relatively low cooling rates (approx. 10 °C/min), by allowing the press plates to slowly cool down through a controlled water circulation.

Rheological characterization was performed both in shear and non-isothermal elongational flow, using a TA Instruments (New Castle, DE, USA) Ares G2 plate-plate rotational rheometer, and a CEAST (Pianezza, Italy) Rheologic 1000 capillary rheometer, respectively. As regards the characterization in non-isothermal elongational flow, melt strength (MS) and breaking stretching ratio (BSR) were determined, according to the procedures described elsewhere [13].

MS represents the force in the molten filament at breaking, while BSR is the ratio between the drawing speed at breaking and the extrusion velocity, in runs in which the drawing velocity increases with steady acceleration. The capillary has a length-to-diameter (*L*/*D*) ratio = 40, and the tensile apparatus was located 25 cm below the capillary die [14]. Reproducibility of all of the results was good (±5%).

Anisotropic samples were prepared via film blow, carried out in a Brabender (Duisburg, Germany) single screw extruder (*D* = 19 mm, *L*/*D* = 25; thermal profile 180/200/220/240 °C; screw speed 60 rpm) equipped with a film blowing unit. The blow-up ratio (BUR) was about 4, while the draw ratio (DR) was 5. Samples where then cut off from the obtained films in the machine direction (MD).

Morphological characterization was performed via scanning electron microscopy (SEM) on nitrogen-fractured samples, using a FEI (Hillsboro, OR, USA) Quanta 200F apparatus. Particle size distribution was analyzed using Leica (Wetzlar, Germany) QWin analysis software; in particular, average particle diameters were calculated by using the following formula (Equation (1)):

(1) $$D=\frac{\sum_{i=1}^{n}n_{i}D_{i}}{\sum_{i=1}^{n}n_{i}}$$

where *D_i_* is the diameter of the *i*-th particle, and *n_i_* is the number of particles having *D_i_* as diameter.

Mechanical characterization was performed in tensile mode on both the isotropic (i.e., sheets) and the anisotropic (i.e., oriented films) samples, using a Instron (Norwood, MA, USA) 3365 universal machine, according to ASTM D882 (grip distance 50 mm, crosshead speed 50 mm/min). The reproducibility of the results was satisfactory (±7%).

Crystallinity degree of the investigated materials was assessed by differential scanning calorimetry (DSC), using a Shimadzu (Kyoto, Japan) DSC-60 equipment, under nitrogen flow (temperature from 30 to 250 °C, at 10 °C/min).

## 3. Results and Discussion

As already discussed, the work is aimed at assessing the effects of the elongational flow, with particular regard to possible “compatibilizing” action. However, in order avoiding possible misinterpretation about this issue, it is worth briefly discussing what “compatibilization” means, which properties should be monitored to evaluate the compatibilizing effects, and taking into account if other causes which may affect the unequivocalness of this comparison (such as different crystallinity degree) are present. It could be stated that compatibilization is a morphology modification, which leads to the improvement of some macroscopical properties. In particular, the elongation at break is known to be particularly susceptible to morphology changes [15,16].

Of course the elongation at beak depends, in its turn, on several properties of the material itself, such as orientation and crystallinity degree, in the case of pure polymers. In the case of polymer blends, it depends on the matrix orientation, crystallinity of the two phases, size and shape of the second phase, orientation of the second phase, adhesion between the phases. Usually, the elongation at break decreases (while the elastic modulus and the tensile strength increase) upon increasing the orientation and the crystallinity; the degree of adhesion also enhances the mechanical properties. With regard to shape and size, it can be stated that the mechanical (tensile) properties of a blend improve upon increasing the surface-to-volume ratio of the second-phase particles. Furthermore, the orientation of the second phase can also increase the elongation at break, since there are more chances for the matrix macromolecules to creep over the elongated particles. In this context, compatibilization can be regarded as the improvement of at least a mechanical property; in particular, elongation at break was chosen, since it is the most susceptible to morphological changes.

**Figure 1:**
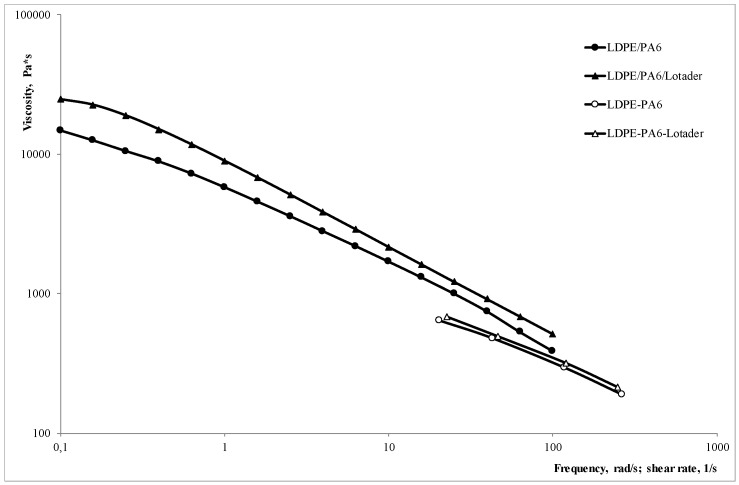
Flow curves of the compatibilized and uncompatibilized blend (solid points have been measured in the rotational rheometer, while open points in capillary viscometer). LDPE: low density polyethylene.

Figure 1 reports the rheological curves of the investigated systems, obtained using both a plate–plate rotational rheometer and a capillary viscometer. These show lower values in comparison to those measured through the rotational rheometer, therefore the Cox–Merz rule is not valid here.

This is not very surprising since such behavior has already been observed in previous works on heterogeneous systems, comparable to those investigated here [17,18,19,20]. In particular, this discrepancy is mainly due to the convergent flow at capillary inlet, which orients the dispersed particles along the flow direction, thus reducing the resistance to the flow.

**Figure 2:**
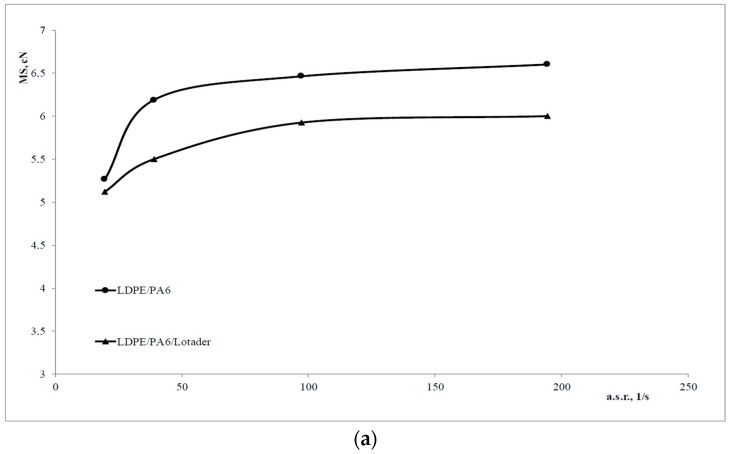

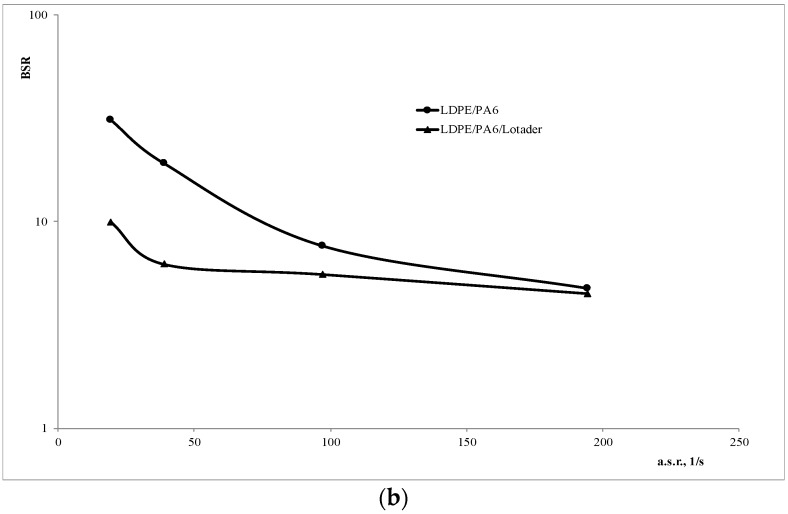
Melt strength, MS, (**a**) and breaking stretching ratio, BSR, (**b**) of the uncompatibilized and compatibilized blends.

Non isothermal elongational flow rheological tests allowed measuring the MS and the BSR, which are shown in Figure 2a,b, respectively.

BSR (i.e., melt deformability) is significantly lower in the compatibilized blend, and this is in agreement with the higher viscosity found (Figure 1); at the same time, MS is also lower, while a higher value may be expected, based on the behavior of the pure polymers where MS is typically a “mirror image” of the BSR. However, this is not a contradiction, but can be explained considering the aforementioned, significantly lower deformability, that leads to premature melt rupture.

**Figure 3:**
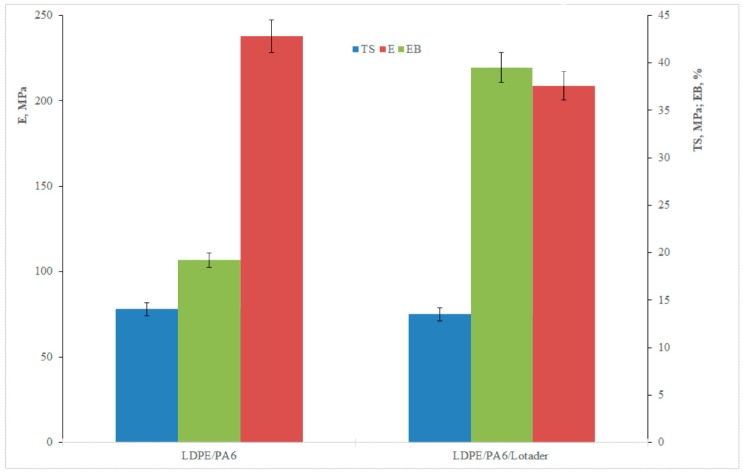
Tensile properties of the isotropic blends. Elastic modulus, E; tensile strength, TS; elongation at break, EB.

Figure 3 shows the tensile properties (elastic modulus, E; tensile strength, TS; elongation at break, EB) of the isotropic samples.

By comparing the properties of the binary and the compatibilized blend, it is easy to observe that the addition of Lotader leads to an increase of the elongation at break and to a fundamentally unchanged tensile strength. On the other hand, the elastic modulus decreases because of the significantly lower value of this property with reference to the Lotader itself and, partially, to a marginal reduction of the crystallinity, as shown in the following Table 1, where the melting enthalpies of the PA6 and LDPE phases are reported.

**Figure 4:**
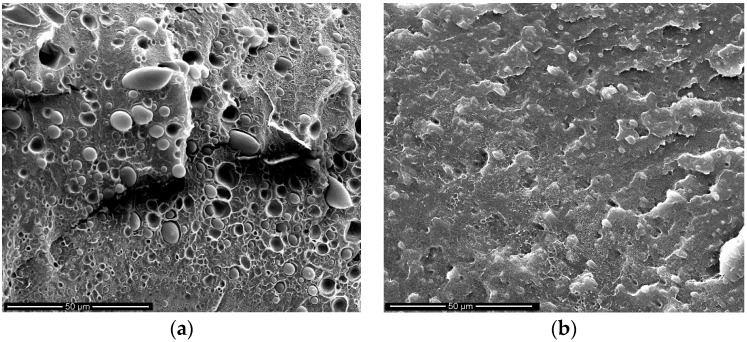
SEM images of the isotropic samples: binary blend (**a**); compatibilized binary blend (**b**).

In order to better explain the actual behavior of the investigated systems, SEM morphological analysis was performed on both the isotropic, uncompatibilized (Figure 4a) and compatibilized (Figure 4b) samples.

The uncompatibilized blend shows a heterogeneous morphology, with spherical particles of the dispersed phase and with a very poor adhesion. On the contrary, the ternary blend shows a more homogeneous morphology, with the two phases appearing almost undistinguishable and then with a good adhesion. The effect of this morphology directly reflects on the better mechanical properties already reported.

**Figure 5:**
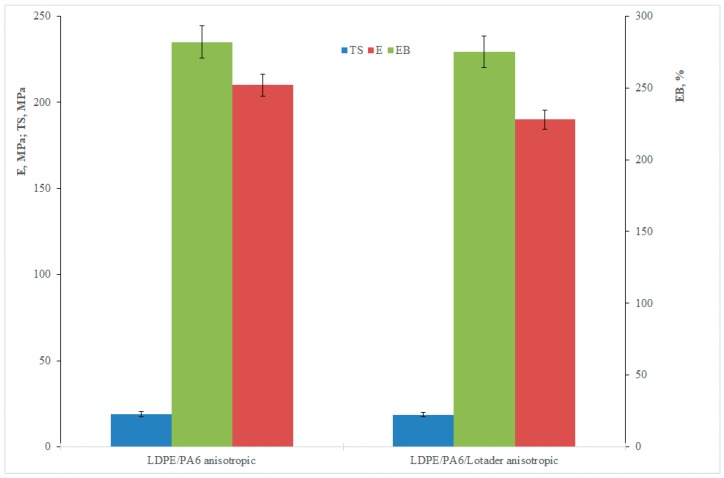
Tensile properties of the anisotropic blends.

With regard to the mechanical properties of the anisotropic samples, these are reported in Figure 5.

**Figure 6:**
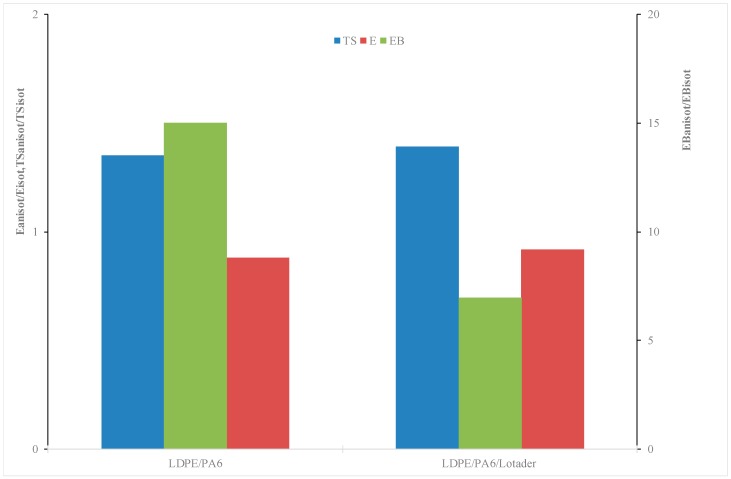
Dimensionless (anisotropic vs. isotropic) tensile properties of the investigated systems.

A first result is the confirm that the ternary (compatibilized) blend has a slightly lower modulus in comparison to the binary blend, in agreement with the evidences already found for the isotropic samples. On the other hand, unexpectedly, the values of the elastic modulus of the anisotropic film are lower than the respective of the isotropic samples. This can be further seen by considering the dimensionless plots (i.e., the ratio between a specific average tensile property of the anisotropic, oriented film and the corresponding average property of the isotropic sheet), Figure 6, which allows estimating the actual effect of the orientation: indeed, it can be observed that the elastic modulus decreases (left axis).

Of course, the orientation should give rise to some improvement of the elastic modulus along the drawing direction, due to the orientation of the macromolecules. However, the decreases found here may be explained considering the crystallinity of the two phases in the anisotropic samples, see Table 2, in comparison to the isotropic ones, see Table 1. More specifically, the crystallinity of the two phases in the anisotropic film sample is lower than that in the isotropic sheet sample. The lower crystallinity gives rise, of course, to lower values of the elastic modulus. Such lower crystallinity in the anisotropic films can be easily explained considering the very high cooling rates in the film blowing operation and the very low thickness of the films (about 80 micron) in comparison to the isotropic, compression-molded sheets (about 600 micron). The high cooling rate and the subsequent little time available to the crystallization, give rise to films with lower crystallinity if compared to the thicker sheets, especially for the PA6 phase, which crystallization rate is lower than that of the LDPE.

**Figure 7:**
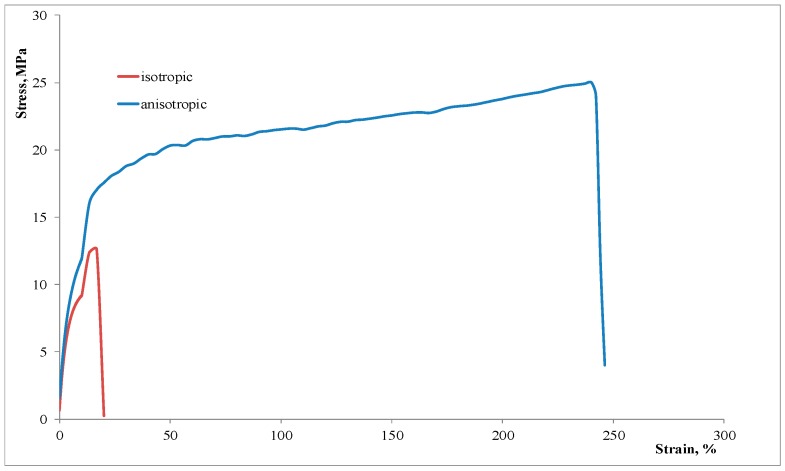
Stress–strain curves of binary blend samples (isotropic vs. anisotropic).

With regard to the mechanical behavior, the properties at break of these oriented, anisotropic film samples show some unexpected trends. In particular, a strongly unexpected result is the behavior at break, since the samples become ductile (or more ductile) after undergoing orientation during the film blowing process. The elongation at break, see Figure 6, increases approximately of one order of magnitude (right axis) and—much more important—the behavior at break of the binary blend is even better than that shown by the ternary, compatibilized blend. In fact, a decrease of the elongation at break was expected, induced by the orientation [20,21]. In particular, as further observable from the stress–strain curves shown in Figure 7, the oriented binary blend film sample undergoes a brittle–ductile transition in comparison to the unoriented, isotropic sample.

**Figure 8:**
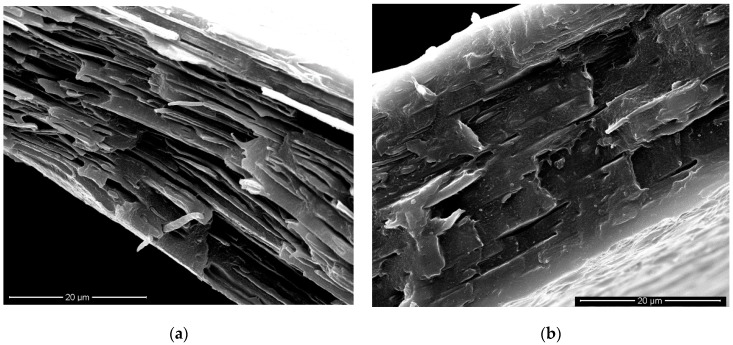
SEM images of the anisotropic samples: binary blend (**a**); compatibilized binary blend (**b**).

The SEM micrograph of the anisotropic sample (Figure 8a) shows that the polyamide-phase dispersed particles are deformed (cylinder-shaped rather than spherical) and preferentially oriented along the drawing (machine, MD) direction.

The dimensions of these anisotropic particles are about 8.5 µm in length and about 0.6 µm in diameter (average values), with an average length-to-diameter ratio of about 13.5. The average surface-to-volume ratio was then calculated to be about twofold if compared to the spherical particles of the isotropic samples. Then, the fragile–ductile transition of the oriented film can be attributed to the increase of the contact area between the two phases and to the subsequent improvement of the stress transfer between them. In fact, the elongated macromolecules of the PE matrix are deformed like in the neat polymer and the elongated PA6 particles, with a relatively high contact area and oriented in the same direction of the PE macromolecules, do not represent a defect (differently from the isotropic sample) thanks to the higher contact surface which mitigates the effects related to the weak adhesion [10]. Since the elongation at break is a macroscopic property that strongly depends on the quality of morphology, it may be thus stated that the orientation through elongational flow has effects which can be compared with those from a “classic” compatibilization.

Of course, also the blend compatibilized with Lotader shows an increase of the EB in comparison to the isotropic compatibilized blends. This can be explained (in similarity to the case of the binary blend) by considering an improvement of the morphology: even in this case, there is an increase of the contact area between the two phases. Furthermore, it should be observed from the dimensionless plots (Figure 6) that the changes in the properties, due to the orientation, are comparable or even higher in the binary blends, if compared to the compatibilized blend. This further suggests that the processing method, and in particular the orientation achieved thanks to the elongational flow, can lead to improvements which are normally achieved only by adding a compatibilizer. On the other hand, the anisotropic blend did not gain significant improvements from the presence of the compatibilizer; this can be attributed to the modifications in the morphology where, as previously pointed out, the compatibilized anisotropic blend (Figure 8b) shows smaller cylinder-like domains in comparison to the binary (uncompatibilized) anisotropic blend (Figure 8a).

## 5. Conclusions

The effect of the orientation on the mechanical properties of a LDPE/PA6 incompatible blend was studied, with particular concern on the comparison with the effects obtained by adding an ethylene-glycidyl methacrylate compatibilizer. The experimental results clearly show that the properties of a film based on a LDPE/PA6 blend are practically identical (if not better) in the binary, oriented film if compared to the compatibilized one. Moreover, the oriented LDPE/PA6 blend showed a brittle-to-ductile transition induced by the orientation, with significantly higher values of the elongation at break if compared not only to the isotropic, uncompatibilized blend, but also the compatibilized one. This means that the processing method, and in particular the orientation achieved thanks to the elongational flow, allowed obtaining improvements which are normally achieved only through adding a compatibilizer. Therefore, taking the elongation at break as a macroscopic compatibilization index (since it is a macroscopic property that strongly depends on the quality of morphology), the orientation through elongational flow has effects that can be compared with those from a chemical compatibilization.

## Figures and Tables

**Table 1:** Melting enthalpies (obtained from differential scanning calorimetry (DSC) analysis) of the PA6 and LDPE phases.

System	*ΔH_m_* PA6, J/g	*ΔH_m_* LDPE, J/g
LDPE/PA6	41.1	83.1
LDPE/PA6/Lotader	39.7	81.2

**Table 2:** Melting enthalpies of the PA6 and LDPE phases (anisotropic samples).

System	*ΔH_m_* PA6, J/g	*ΔH_m_* LDPE, J/g
LDPE/PA6	32.1	79.2
LDPE/PA6/Lotader	29.3	71.1
